# Fatty Acid and Tocopherol Composition of Pomace and Seed Oil from Five Grape Varieties Southern Spain

**DOI:** 10.3390/molecules27206980

**Published:** 2022-10-17

**Authors:** Yolanda Carmona-Jiménez, Jose M. Igartuburu, Dominico A. Guillén-Sánchez, M. Valme García-Moreno

**Affiliations:** 1 Department of Analytical Chemistry, Faculty of Science, Wine and Food Research Institute (IVARO), University of Cadiz, Campus Universitario de Puerto Real, 11510 Puerto Real, Cádiz, Spain; 2 Department of Organic Chemistry, Faculty of Science, University of Cadiz, Campus Universitario de Puerto Real, 11510 Puerto Real, Cádiz, Spain

**Keywords:** winery by-products, vegetable oils, vitamin E, atherogenicity index (AI), thrombogenicity index (TI), ratio between hypo- and hypercholesterolemic fatty acids (H/H), vitamin E activity

## Abstract

Grape pomace and seeds are important winemaking by-products. Their oils are rich in bioactive compounds such as fatty acids and tocopherols. We have characterized oils from both by-products from five Spanish grape varieties (Palomino Fino, Pedro Ximénez, Muscat of Alexandria, Tempranillo and Tintilla de Rota). A high content of UFAs was found in all the analyzed samples. Grape pomace oils generally had the same oleic acid (PUFA_ω-6_) content as seed oils, and lower PUFA contents; they also had a markedly higher linolenic acid (PUFA_ω-3_) content, improving the PUFA_ω-6_/PUFA_ω-3_ ratio. All the oil studied show good indicators of nutritional quality: low values of the atherogenicity (0.112–0.157 for pomace, 0.097–0.112 for seed) and thrombogenicity indices (0.30–0.35 for pomace, 0.28–0.31 for seed) and high values of the relationship between hypo- and hypercholesterolemic fatty acids (6.93–9.45 for pomace, 9.11–10.54 for seed). Three tocopherols were determined: α-, γ- and δ-tocopherol. Pomace oils have higher relative contents of α- and δ-tocopherol, whereas seed oils have higher relative contents of γ-tocopherol. A significantly higher content of total tocopherols has been found in pomace oil; it is higher in the oils from red varieties of pomace (628.2 and 706.6 mg/kg by-product), and in the oils from pomace containing stems (1686.4 mg/kg by-product). All the oils obtained can be considered as a source of vitamin E, and their consumption is beneficial for health.

## 1. Introduction

The interest in identifying and using unconventional vegetable oils that are rich in compounds with high biological value has increased in recent years [1,2,3]. Grape seed oil is a product with great potential for use in culinary, pharmacological or cosmetic applications due to its unique composition [3,4,5,6]. 

Grape seeds contain 10–20% oil, and they are mainly obtained from pomace, which is obtained by the grape-pressing procedure in juice and wine production. In fact, grape pomace is the most abundant by-product generated in the wine industry, and it is composed of grape seeds, skin, and sometimes stems. The usual process of obtaining grape seed oil involves the separation of the grape seeds from the rest of the pomace and the subsequent extraction of the oil with hexane [7,8,9]. The amount of oil obtained and its composition strongly depend on factors such as the grape variety and its growing conditions [10,11,12,13,14]. Grape seed oil is rich in unsaturated fatty acids, mainly linoleic acid, and vitamin E [15,16,17]. Linoleic acid, together with linolenic acid, is one of the more essential fatty acids that cannot be synthesized by the human metabolism. Grape seed oil contains, depending on the grape variety, 61–73% linoleic acid (C18:2), 14–25% oleic acid (C18:1) and 0–0.6% linolenic acid (C18:3) [18]. Hence, grape seed oil is composed of 85–90% unsaturated fatty acids. 

Numerous studies have highlighted the significant roles of essential fatty acids in many biochemical pathways, including their cardioprotective effect due to their considerable antiatherogenic, antithrombotic, anti-inflammatory, antiarrhythmic and hypolipidemic effects [19,20,21]. These compounds have a complex influence on concentrations of lipoproteins, the fluidity of biological membranes, the function of membraned enzymes and receptors, the modulation of eicosanoids production, blood pressure regulation, and the metabolism of minerals [20] and cholesterol [22,23,24]. 

The fatty acids profile may be an indicator of functional qualities of oil obtained from vinification by-products. Thus, the unsaturation ratio (USFA/SFA), an indicator of the proportion of unsaturated fatty acids related to saturated fatty acids, is used to evaluate the nutritional value of the lipid fraction of food, and it is also used to determine indexes for recurrent cardiovascular disease syndromes such as atherogenicity or thrombogenicity [24]. In nutrition, high values of this ratio are desirable since they express the effect of FAs on cholesterol metabolism. Other indices related to the cardiovascular health of the dietary fat are the atherogenicity index (AI), the thrombogenicity index (TI) and the ratio between hypo- and hypercholesterolemic fatty acids (H/H). AI is a measure of the risk of obstruction of the arteries; TI is a measure of the risk of producing thrombus or blood clots [25], and the H/H ratio is related to cholesterol metabolism. Oils with high values of this ratio are beneficial for health since they contribute positively to the improvement of cardiovascular health.

Grape seed oil is also one of the richest natural sources of vitamin E [21,26]. Vitamin E is a fat-soluble micronutrient formed by a chromanol ring and a hydrophobic side chain. Vitamin E is the generic term applied to a group of eight vitamers: four tocopherols (α-, β-, γ- and δ-tocopherol), which differ in both the number of methyl substituents and their position on the phenolic ring, and their four corresponding tocotrienols, which differ from tocopherols due to the presence of three double bonds in their side chain. Each form of Vitamin E has its own biological activity, with α-tocopherol considered to be one of the most active [27,28,29]. Vitamin E is the most effective natural lipid-soluble antioxidant [30]. Thus, it protects cytoplasmic membranes from oxidation and low-density lipoproteins from dangerous lipid peroxidation processes [31]. Vitamin E is also associated with the prevention of coronary heart disease, atherosclerosis, cancer, diabetes, Parkinson’s disease, Alzheimer’s disease and impaired immune function, amongst others [32,33,34]. 

Although information about the content of fatty acids and tocopherols in grape seed oils from different regions has been reported [4,10,12,35,36,37,38,39], as well as their nutritional indicators [22,23,24], no results have been published on the varieties that are studied in this work: four Spanish varieties grown in Andalusia, Palomino Fino, Pedro Ximénez, Moscatel de Alejandría and Tempranillo, together with a fifth typically Andalusian variety, Tintilla de Rota. Likewise, in the consulted bibliography, we found very little information on the fat-soluble composition [6,40,41,42,43], and almost nothing on the nutritional details of grape pomace oils [24], not finding any work related to the pomace oil of the five grape varieties studied in this work.

The Palomino Fino, Pedro Ximénez and Moscatel de Alejandría varieties are grape varieties widely used for the production of typical wines from the southern area of Spain (Andalusia). The traditional dry fortified wines of the Protected Denomination of Origin (P.D.O.) Jerez-Xérèz-Sherry and P.D.O. Manzanilla-Sanlúcar de Barrameda are made mainly with the Palomino Fino variety, the fortified wines of the P.D.O. Montilla-Moriles are made mainly with the Pedro Ximénez variety, and with the Pedro Ximénez and Moscatel de Alejandría varieties, different types of white wines are made, with the P.D.O. Malaga and Sierra de Malaga wines being the most traditional among Andalusian wines. Likewise, with the grapes of the Pedro Ximénez and Moscatel de Alejandría varieties, sweet wines are produced in all the indicated protected designations of origin. The Tempranillo variety is the red variety most used for the production of red wines in Spain, and the Tintilla de Rotay variety is a red variety native to the sherry area [44], with which traditional sweet red wines are made, as well as other red wines, and it is very appreciated at an organoleptic level for its uniqueness.

For this reason, the objective of this study was the characterization of the main fat-soluble compounds (fatty acids and tocopherols) of the seed oils and grape pomace of the varieties Palomino Fino, Pedro Ximénez, Moscatel de Alejandría, Tempranillo and Tintilla de Rota, and their relationships with the main nutritional indices (AI, TI, H/H and vitamin E activity), with the aim of determining the functional qualities of these oils and whether they can be considered healthy and recommended for human consumption, such as olive oil or sunflower oil.

## 2. Results and Discussion

### 2.1. Oil Content

The oil contents of the analyzed pomaces and seeds, expressed as the percentage of oil in the dried by-product, are shown in Table 1. 

In relation to the oils obtained from the grape pomace, the extraction yields were between 3.81% and 9.73% (g oil by 100 g of by-product), and the oils obtained from the grape seed were between 12.46 and 22.52%. In pomace oils, the red varieties gave slightly superior values to the white ones, with the MA variety showing particularly low values. These values are similar to those found by El Gengaihi et al. [42] in the pomace from some grapes grown in Egypt, and by Gülcü et al. [43] in pomace from the Merlot grape. These authors reported that the grape pomace contained between 3.1% and 9.5% oil, and 4.75% and 8.90%, respectively. This lower extraction yield per gram of matter extracted from the pomace is to be expected, since the oil that is extracted comes mainly from grape seed, and the seed content in the pomace is between 38% and 52% of the total matter. In pomace oils, the red varieties gave slightly superior values to the white ones, with the MA variety showing particularly low values. These values are similar to those found by El Gengaihi et al. [42] in the pomace from some grapes grown in Egypt, or Gülcü et al. [43] in the pomace from Merlot grapes. These authors reported that grape pomace contained between 3.1% and 9.5% oil, and 4.75% and 8.90%, respectively. This lower extraction yield per gram of matter extracted from the pomace is to be expected, since the oil that is extracted comes mainly from the grape seed, and the seed content in the pomace is between 38% and 52% of the total matter. 

On the other hand, the extraction yields from the grape seeds were between 12.46% and 22.52%. Other authors have reported that grape seeds contain 3.95% and 20.71% oil depending on the grape variety [4,17,35,36,45]. Wen et al. [35] found values between 13.71% and 15.92% for the Chardonnay, Merlot and Cabernet Sauvignon varieties, with the Merlot variety having the highest value and the Chardonnay variety having the lowest value; Fernandes et al. [4] obtained lower for Portugese red varieties, with the Marufo variety having the lowest value (3.95%), and the Touriga Francesa variety having the highest value (12.40%); Tangolar et al. [17] analyzed grape seed oil from nine grape varieties, finding values between 16.71% (Salt creek) and 7.48% (Horoz karasi); Demirtas et al. [36], studying the grape seed oil of seven native Turkish cultivars, found higher oil content values, with values between 15.30% and 20.71%; Bada et al. [45] characterized six grape seed oils from varieties grown in different areas of Spain, and found values between 7.57% (Tempranillo from Toro) and 13.89% (Albarin from Cangas). The seeds of the grape varieties studied in this study have oil yields similar to those found in the bibliography, with the PF and PX varieties showing values somewhat higher than 20%, and the TM variety a slightly lower percentage (12.46%).

### 2.2. Fatty Acid Composition

Table 2 lists the eleven different fatty acids that were detected and quantified in the pomace and seed grape oils investigated. Six saturated fatty acids (SFAs) (lauric acid (C12:0), myristic acid (C14:0), palmitic acid (C16:0), stearic acid (C18:0), arachidic acid (C20:0) and behenic acid (C22:0)), three monounsaturated fatty acids (MUFAs) (palmitoleic acid (C16:1), oleic acid (C18:1) and erucic acid (C22:1)), and two polyunsaturated fatty acids (linoleic acid (C18:2) and linolenic acid (C18:3)) have been identified. 

The average unsaturated fatty acids (UFA) content was 83.41% in pomace oil and 86.73% in grape seed oil. The monounsaturated fatty acid (MUFA) values obtained were between 16.43% and 20.27%, and the polyunsaturated fatty acid (PUFA) values obtained were between 64.04% and 69.44%, presenting higher grape seed oil values than the grape pomace oil. 

Linoleic acid was the main fatty acid in all samples, as expected, presenting values between 61.37% (PX) and 65.16% (TR) in grape pomace oil and values between 65.55% (PF) and 69.00% (TM) in grape seed oils. The red varieties presented somewhat higher values than the white varieties. Oleic acid is the second most important fatty acid in the samples studied, and the primary MUFA, with values ranging from 16.34% in the pomace oil of the TM variety to 20.13% in the grape seed oil of the PX variety. All of the results obtained are within the range of results given by other authors for different grape seed oils [4,10,17,35,36,45]. Fernandes et al. [4] obtained values for PUFA between 63.64% and 73.53%, and linoleic acid contents between 72.3% (Aragonês) and 63.0% (Tinta Barroca), for seed oils of Portugal; Demirtas et al. [36] found PUFA values between 56.65% and 68.97%, and linoleic acid contents between 68.56% and 56.38%, for different native Turkish grape seed oils; Wen et al. [35] found PUFA values between 63.88% and 77.12%, and linoleic acid contents between 63.52% and 76.77%, for seed oils of grapes grown in China; Bada et al. [45] also found that linoleic acid was the main fatty acid in the seed grape oil analyzed, with values between 68.67% (Tempranillo) and 78.23% (Carrasquín). 

Statistically significant differences were obtained between varieties and between types of oil. As far as varieties are concerned, PF and PX had higher contents of MUFA for both types of oils, i.e., grape pomace oil and grape seed oil, whereas the varieties TM, TR and MA had the highest contents of PUFA. If total unsaturated fatty acids (USFA) are considered, the varieties PX and MA presented the highest total contents in seed oils, whereas PX, PF and TR gave the highest results in pomace oils. 

When comparing the types of oils, i.e., pomace and seed oils, statistically significant differences in the MUFA contents were not found, but they were observed in the PUFA contents. The seed oils were found to have a higher content of PUFAs, mainly due to their contents in linoleic acid, since pomace oils have a significantly higher linolenic acid content, with values between 0.90% in the PX variety and 4.03% in the MA variety (both white varieties). The average values of linolenic acid found in the bibliography for other grape oils are around 0.30–0.4%, values similar to those found in the grape seed oils analyzed in this study [4,5,10,17,23,24,35,36,45]. That is, the percentage content of linolenic acid found in grape pomace oil is 3 to 10 times greater than in the corresponding grape seed oils. Very few vegetable oils contain linolenic acid in appreciable amounts. Orsavova et al. [20] found the highest contents, between 1.2% and 1.6%, of linolenic acid in wheat germ, rapeseed and olive oil, among the other 11 oils studied. Bondioli et al. [46] explained that only soy and rapeseed oils, among the common commercial oils, contain up to 10% linolenic acid. This acid is an essential fatty acid, being the main representative of the PUFA_ω-3_ acids. It is known that a balance between PUFA_ω-6_ and PUFA_ω-3_ intake maximizes the benefits that PUFAs provide [20]. In western countries, a high consumption of oil rich in PUFA_ω-6_ is very usual, but so is a low consumption of PUFA_ω-3_, which can unbalance the relation of PUFA_ω-6_/PUFA_ω-3_ aforementioned. For this reason, the search for oils with higher PUFA_ω-3_ contents is increasingly common [46]. The amount of linolenic acid found when the oil is extracted using the whole grape pomace is significantly higher, making it more beneficial in terms of the PUFA_ω-6_/PUFA_ω-3_ ratio. Therefore, it can be concluded that grape pomace oils generally contain the same contents of oleic acid as the grape seed oils, and lower contents of PUFA, although the differences were less than 5% in all cases, whereas pomace oils had a markedly higher content of linolenic acid. The variety PX should be highlighted due to its higher unsaturated fatty acid content in all types of oil.

It is known that a high value of fatty acids in oils may be associated with the greater ease of oxidation, giving rise to problems of oxidation and the deterioration of the oils [12]. For this reason, COX values have been calculated to determine the oxidative stability of the grape oils studied. Table 3 shows these values together with other nutritional indicators of the grape oils studied: USFA/SFA ratio, PUFA/SFA ratio, H/H, AI and TI. 

The pomace and grape seed oils studied show COX values similar to those previously reported by other authors for grape seed oils [39,47]. These values are higher than those of other vegetable oils, such as olive oil or palm oil, similar to those of sunflower oil [48] or soybean oil [49], and lower than those of flaxseeds oil [50]. 

As previously mentioned, the fatty acids profile may be an indicator of functional qualities of oil obtained from vinification byproducts. The grape pomace oil samples had USFA/SFA ratio values between 4.78 (TM) and 6.04 (PX), and the grape seed oil samples had values between 6.21 (PF) and 6.92 (MA). Another important index is the PUFA/SFA ratio. The British Department of Health considers the recommended PUFA/SFA ratio is a good indicator of the nutritional value of dietary fat. In the human diet, values for this ratio greater than 0.45 are recommended [51]. As shown in Table 3, all the grape oils analyzed were in agreement with this requirement. For this ratio, the grape pomace oil samples presented values between 3.82 (MA and TM) and 4.73 (PX), and the grape seed oil samples presented values between 4.78 (PF) and 5.50 (MA). The values obtained for both ratios are similar to those found in the bibliography consulted for grape pomace and grape seed oils [24,39].

The values of AI and TI ranged from 0.112 (PX) to 0.157 (TM) and from 0.30 (PX and MA) to 0.35 (TM), respectively, for grape pomace oil, and from 0.097 (PX) to 0.112 (TR) and from 0.28 (PX and MA) to 0.31 (PF), respectively, for grape seed oil. These AI and TI values are similar or superior to those found by other authors in seed oils from white and red grapes [23,24]. These values are similar to those found in sunflower oil, somewhat lower than those found in olive oil, and lower than those found in palm and soybean oils [48,49]. In general, the seed oils studied show values somewhat lower than those of pomace oil for both indices. However, in view of the results obtained, it can be considered that these oils are just as healthy, and can be as highly recommended for human consumption, as olive oil or sunflower oil. In our research, the H/H values of grape pomace oil are lower than those of grape seed oil. The grape pomace oils range from 6.93 for the TM variety to 9.45 for the MA variety, and for the grape seed oils, they range from 9.11 for the TR variety to 10.54 for the PX variety. The H/H values obtained for all studied oils are similar to those found by Ferreira et al. in grape pomace oil [24], and lower than those found by Dimíc et al. for grape seed oils [23]. These H/H values are also higher than those reported in the bibliography by Hashempour-Baltork et al. [22] in olive and sesame oils, and less than in flaxseed oil. 

### 2.3. Tocopherol Composition

Three tocopherol forms were determined: α-tocopherol, γ-tocopherol and δ-tocopherol. β-tocopherol was not detected in any oil (Table 4). The most abundant tocopherol in all the samples was α-tocopherol, followed by γ-tocopherol. 

Total tocopherol contents for grape pomace oils were obtained in the range from 228.9 to 1686.4 mg/kg pomace oil. In this case, an average of 83.9% corresponds to α-tocopherol, 10.5% to γ-tocopherol and 5.6% to δ-tocopherol. Huge differences were found between the total tocopherol contents of the different grape pomace varieties. The PF pomace oil had the highest content of tocopherol, followed by the oils obtained from the two red varieties analyzed, TM and TR, between which statistically significant differences were not found. The pomace oils with the lowest tocopherol contents were the MA variety, and finally the PX pomace oil, with significant differences observed between the two.

On the other hand, the total tocopherol contents of grape seed oils ranged from 196.2 to 248.0 mg tocopherol/kg oil, of which an average of 73.6% corresponds to α-tocopherol, 23.6% to γ-tocopherol and 2.8% to δ-tocopherol. The seed oil samples from the PX and MA varieties were the only ones in which δ-tocopherol was not detected. In the case of the grape seed oils, all varieties showed similar results for the total tocopherol content, although statistically significant differences were found between the PF seed oil, with the highest content, and the MA and TR seed oils, with the lowest contents. The results obtained for grape seed oils are in the same order as those reported in the literature for grape seed oils of different origins. Wen et al. [35] obtained total tocopherol values between 62.0 and 175.9 mg/kg oil for different grape varieties grown in China, Demirtas et al. [36] found total tocopherol contents between 102.3 and 305.4 mg/kg oil in native grape varieties grown in Turkey, and Dimić et al. [23] found red seed grape values ranging from 6.33 to 7.96 mg 100 g^−1^, and white seed grape values from 0.95 to 2.63 mg 100 g^−1^. 

The comparison of the results for pomace oils with those for the seed oils of the same varieties (Table 4) shows marked differences both in the quantity and composition of tocopherols. In general terms, the pomace oils have higher relative contents of α-tocopherol and δ-tocopherol, whereas seed oils have higher relative contents of γ-tocopherol.

As can be observed, the total amounts of tocopherols obtained when the whole pomace is extracted are much greater than those in seed oils, except for the PX oils, in which case the results are similar. The oil of PF pomace is almost seven times richer in tocopherol than the seed oil of the same variety. The pomace oil of the red varieties analyzed, TM and TR, had three times the tocopherol contents of their analogous seed oils, whereas the MA variety had double the content. It is significant that the greatest differences were found in the PF oils, since the pomace of this variety is the only one that contained stems. It has been shown that when the oil is extracted with the whole grape pomace, generally, it has a far higher tocopherol content, which can be up to seven times higher than in the seed oil. The tocopherol content found in grape pomace oil is much higher than the tocopherol content found in many of the most consumed vegetable oils. For example, [52] obtained total tocopherol contents ranging from 193 to 371.9 mg/kg in different olive oils, whereas Yuan et al. [53] found total tocopherol contents of 399 mg/kg in soybean oil, 360 mg/kg in corn oil and 177 mg/kg in camellia oil. Aksoz et al. [54] found, among others, 538.1 mg/kg in sunflower oil, 121.6 mg/kg in red palm oil, 293.4 mg/kg in almond oil, 69.8 mg/kg in peanut oil, 427.5 mg/kg in sesame oil and 742.0 mg/kg in corn oil. Therefore, compared to the aforementioned data, the tocopherol contents found in the PF pomace oil (1570.1 mg/kg), in the TM pomace oil (543.5 mg/kg) and in the TR (647.4 mg/kg) are very remarkable. These results are in agreement with those obtained in other studies with grape bagasse (skin and stem) and grape stem [26,40]. Göktürk Baydar and Özkan [40] observed that tocopherols are found not only in the seeds, but also in the skin and stem. Thus, they obtained higher values of total tocopherol in pomace and bagasse than in grape seeds. Tangolar et al. [26] observed that pomace and especially bagasse and grape stalk extracts contained higher tocopherol contents than the seed extracts.

The vitamin E activity of grape oil samples ranged between 1574.6 mg/kg of pomace oil of the PF variety and 122 mg/kg of seed oil of the TR variety. With the exception of pomace oil of the PF variety, all oils show vitamin E activity values similar to those reported by other authors for grape seed oils.

According to the National Research Council [4], the recommended daily intake of α-tocopherol is 15 mg, considering that an oil is a “source of vitamin E” when it contains at least 0.5 mg of α-tocopherol equivalent per 10 g of oil. According to the data in Table 4, all the oils obtained, and especially those from grape pomace, can be considered as a source of vitamin E, since they all contain more than 0.5 mg of α-tocopherol equivalent per 10 g of oil, so their consumption is beneficial for health. For the same variety, pomace oils have higher values of equivalent vitamin E than seed oils, and we emphasize the PF variety, due to its high α-tocopherol composition.

## 3. Materials and Methods

### 3.1. Samples

Five varieties were studied: three white varieties, Palomino Fino (PF) and Pedro Ximénez (PX) and Muscat of Alexandria (MA), and two red varieties, Tempranillo (TM) and Tintilla de Rota (TR). 

This study was carried out with grape pomace collected from a vineyard located in the Jerez area, in southwestern Spain, in the 2014 harvest. To limit the influence of external factors and allow a better comparison between the results, all the samples shared the same geographic area and winemaking practices. The pomace was formed from grape seeds and skin, and only the PF variety contained stems. The grape seeds were obtained by hand separation from the pomace. 

All the samples were collected just after pressing and they were then dried in a climatic chamber at 40 °C, 10% relative humidity and darkness until constant weight [55]. Then, the samples were triturated, with a conventional beater, and sieved until a homogenous sample was obtained. 

### 3.2. Chemicals

Tocopherol standards, n-hexane and isopropanol were supplied by Merck (Darmstadt, Germany). Standards of fatty acids, anhydrous methanol and 3N methanolic HCl solution were purchased from Sigma- Aldrich (St. Louis, MO, USA). All solvents were of HPLC grade.

### 3.3. Oil Extraction Procedure

Ten grams of by-product, pomace or grape seed, were placed into a Soxhlet extraction thimble. Samples were extracted using n-hexane according to AOAC method no. 920.85 [56]. The resulting extracts were evaporated to dryness on a rotary evaporator at a temperature not exceeding 40 °C. The oils were weighed, and the mass percentage of oil extracted was calculated. Finally, oils were stored under nitrogen at −20 °C prior to analyses. All extractions were carried out in duplicate. 

### 3.4. Fatty Acid Determination and Calculated Oxidizability

An analytical method for fatty acids was employed, and this included derivatization of the fatty acids to methyl esters and analysis by gas chromatography [57]. A quantity of 10 mg of oil was transferred to a hydrolysis tube according to the protocol described by Gómez et al. [58] with slight modifications. Samples were derivatized with 2 mL of 1.75 M HCl in dry methanol for 18 h at 60 °C under dry nitrogen. The methanol was evaporated under a stream of nitrogen and the methyl esters were then extracted twice with 3 mL of hexane and 2 mL of water. The combined organic layers were dried under a stream of nitrogen and the resulting solid was dissolved in 600 μL of hexane prior to analysis by gas chromatography. A GC-MS Varian Saturn 2200 system equipped with a Varian Factor Four Capillary Column VF-Waxms (30 m × 0.25 mm with a 0.25 μm film thickness) was used for fatty acid analysis. The injection volume was 1 μL and helium was used as the carrier gas at a flow rate of 1 mL/min. The injector temperature was 250 °C and the oven temperature was 150 °C, with a temperature program reaching up to 220 °C at 4 °C/min. Heptadecanoic acid (C17) and heneicosanoic acid (C21) were used as internal standards. Identification was carried out by comparing their retention times to those of the standard peaks. Quantification was expressed as the percentage of total fatty acid methyl ester (FAME). 

Calculated oxidizability (COX) values were determined as the percentage of UFAs (C18) [47,59] (Equation (1)).

(1) COX value=[C18:1 (%)+10.3 × C18:2 (%)+21.6 × C18:3 (%)]/100

Atherogenicity index (AI), thrombogenicity index (TI) and the ratio between hypo- and hypercholesterolemic fatty acids (H/H) were calculated according to Equations (2)–(4), respectively [25],

(2) $$\mathrm{AI}=\frac{\left[C12:0\;(\%)\;+\;4\;\times \;C14:0\;(\%)\;+\;C16:0\;(\%)\right]}{\left({\mathrm{MUFA}\;+\;\mathrm{PUFA}}_{\omega -6}\;{+\;\mathrm{PUFA}}_{\omega -3}\right)}$$

(3) $$\mathrm{TI}=\frac{\left[C14:0\;(\%)\;+\;C16:0\;(\%)\;+\;C18:0\;(\%)\;\right]}{\begin{array}{c} \left(0.5\;\times \;\mathrm{MUFA}\;+\;0{.5\;\times \;\mathrm{PUFA}}_{\omega -6}\;{+\;3\;\times \;\mathrm{PUFA}}_{\omega -3}+\frac{{\;\mathrm{PUFA}}_{\omega -3}}{{\;\mathrm{PUFA}}_{\omega -6}}\right) \end{array}}$$

(4) $$H/H=\frac{\left[C18:1\;(\%)\;+\;C18:2\;(\%)\;+\;C18:3\;(\%)\right]}{\left[C14:0\;(\%)\;+\;C16:0\;(\%)\right]}$$

for this work,

$$\begin{array}{c} {\mathrm{PUFA}}_{\omega -3}\;=\;C18:3\;(\%);\;{\mathrm{PUFA}}_{\omega -6}\;=\;C18:2\;(\%);\\ \mathrm{MUFA}\;=\;C16:1\;(\%)\;+\;C18:1\;(\%)+C22:1\;(\%). \end{array}$$

### 3.5. Tocopherol Determination and Vitamin E Activity

Prior to the analysis, the oil was diluted with hexane and filtered through a 0.45 μm nylon membrane filter. Tocopherols were analyzed on an HPLC system (Waters Alliance 2695, Milford, MA, USA) equipped with a Waters 474 fluorescence detector and a Phenomenex Luna silica column (250 × 4.60 mm, 5 μm). An isocratic mobile phase containing 1.3% isopropanol in *n*-hexane was used according to the methodology described by Lee et al. [60] with some modifications. The flow rate was 1.0 mL/min, the injection volume 10 µL and the column temperature 30 °C. The wavelengths were set at 290 nm for excitation and 330 nm for emission. Tocopherol peaks were identified by comparing their retention times to those of standards. Standard calibration curves were obtained for quantification. 

Vitamin E activity, expressed as mg of α-tocopherol equivalent per kg of oil, was calculated with the following formula [4,61] (Equation (5)):

(5) Vitamin E activity=α-T+0.4×β-T+0.1×γ-T+0.01×δ-T.

### 3.6. Statistical Analysis

All the samples were extracted in duplicate, and each extracted oil was analyzed in duplicate. The results are presented as mean ± standard deviation (SD). Significant differences between sample means were determined by one-way ANOVA, and Fisher’s Least Significant Differences (LSD) test was used to determine different values, with a 95.0% confidence level. The results were processed using *Statgraphics Centurion 19* software (Statgraphics Technologies, Inc., The Plains, VA, USA).

## 4. Conclusions

It has been shown that oil extraction using the whole grape pomace allows oil to be obtained that is the richest in compounds with health benefits. In relation to the content of fatty acids, the general content is very similar between grape seed oil and grape pomace oil. However, it should be noted that a greater amount of linolenic acid (omega-3 acid) is obtained in grape pomace oil, improving the PUFA_ω-6_/PUFA_ω-3_ ratio and making it more balanced. 

With respect to the vitamin E content, we have found a significantly higher content in grape pomace oil. The content increases 2, 3 and up to 7 times when the whole grape pomace is extracted. The tocopherol content of grape pomace oil is much higher than that of most of the usually used vegetable oils. The higher tocopherol levels found were in the red varieties and in the only grape pomace sample that contained stems (PF). 

Therefore, it is advantageous to simplify the process employed to obtain oil by eliminating the step involving the separation of the seeds from the rest of the grape pomace, thus providing an oil with fatty acids and vitamin E contents, and that has great potential for use in the food, pharmacological or cosmetic industries. This way, the benefit of the grape pomace is increased and, at the same time, the waste or byproducts generated by industries such as the wine or juice industries are reduced and re-used.

## Figures and Tables

**Table 1:** Oil yields of pomace oils and grape seed oils (g oil by 100 g of by-product).

	White Grapes			Red Grapes	
	PF	PX	MA	TM	TR
Grape Pomace Oil	6.10 ± 0.13 ^d^	6.71 ± 0.21 ^c^	3.81 ± 0.14 ^e^	8.89 ± 0.04 ^b^	9.73 ± 0.04 ^a^
Grape Seed Oil	22.52 ± 0.63 ^a^	22.32 ± 0.73 ^a^	14.74 ± 0.79 ^b^	12.46 ± 0.30 ^c^	15.89 ± 0.32 ^b^

The results are presented as mean ± standard deviation (*n* = 2). Values followed by the same letter in a row are not different at the 95.0% confidence level (One-way ANOVA with Fisher’s LSD).

**Table 2:** Proportional fatty acid compositions of the by-product oils studied (%).

	White Grapes			Red Grapes	
	PF	PX	MA	TM	TR
Grape Pomace Oils					
C12:0	N.D.	N.D.	0.06 ± 0.03 ^c^	0.22 ± 0.03 ^a^	0.16 ± 0.02 ^b^
C14:0	0.13 ± 0.01 ^d^	0.19 ± 0.01 ^c^	0.34 ± 0.03 ^a^	0.29 ± 0.02 ^b^	0.18 ± 0.01 ^c^
C16:0	9.93 ± 0.15 ^c^	8.44 ± 0.01 ^d^	10.79 ± 0.38 ^b^	11.56 ± 0.14 ^a^	10.89 ± 0.32 ^b^
C16:1	N.D.	0.27 ± 0.02 ^a,b^	0.34 ± 0.01 ^a^	0.34 ± 0.00 ^a^	0.13 ± 0.18 ^b^
C18:0	5.48 ± 0.04 ^a^	4.47 ± 0.01 ^c^	4.74 ± 0.04 ^b^	4.59 ± 0.08 ^b,c^	4.52 ± 0.13 ^c^
C18:1	18.87 ± 0.21 ^a^	17.51 ± 0.13 ^b^	16.32 ± 0.01 ^c^	16.04 ± 0.11 ^c^	16.41 ± 0.20 ^c^
C18:2	63.16 ± 0.34 ^b^	63.14 ± 0.75 ^b^	61.37 ± 0.37 ^c^	64.34 ± 0.14 ^a^	65.16 ± 0.17 ^a^
C18:3	1.52 ± 0.07 ^c^	0.90 ± 0.00 ^d^	4.03 ± 0.01 ^a^	1.69 ± 0.02 ^b^	1.70 ± 0.03 ^b^
C20:0	0.55 ± 0.06 ^b^	0.28 ± 0.04 ^d^	0.81 ± 0.03 ^a^	0.42 ± 0.02 ^c^	0.40 ± 0.01 ^c^
C22:0	0.21 ± 0.01 ^b^	0.17 ± 0.01 ^c^	0.39 ± 0.01 ^a^	0.19 ± 0.01 ^b,c^	0.18 ± 0.02 ^b,c^
C22:1	0.03 ± 0.04 ^a^	N.D.	N.D.	0.05 ± 0.01 ^a^	0.04 ± 0.01 ^a^
SFA	16.30 ± 0.12 ^b^	13.54 ± 0.01 ^c^	17.13 ± 0.46 ^a^	17.27 ± 0.01 ^a^	16.32 ± 0.24 ^b^
USFA	85.65 ± 0.31 ^d^	86.29 ± 0.14 ^b,c^	85.93 ± 0.06 ^c,d^	86.64 ± 0.17 ^a,b^	86.96 ± 0.17 ^a^
MUFA	18.90 ± 0.17 ^a^	17.78 ± 0.11 ^b^	16.66 ± 0.00 ^c^	16.43 ± 0.12 ^c^	16.58 ± 0.01 ^c^
PUFA	64.68 ± 0.42 ^c,d^	64.04 ± 0.75 ^d^	65.40 ± 0.38 ^b,c^	66.03 ± 0.16 ^a,b^	66.87 ± 0.20 ^a^
PUFA_ω-6_/PUFA_ω-3_	41.61	70.04	15.22	38.04	38.26
Grape Seed Oils					
C12:0	N.D.	N.D.	N.D.	N.D.	N.D.
C14:0	0.06 ± 0.00 ^b^	0.06 ± 0.01 ^b^	0.08 ± 0.01 ^a^	0.05 ± 0.01 ^b^	0.06 ± 0.00 ^b^
C16:0	8.30 ± 0.17 ^c^	8.15 ± 0.10 ^c^	8.66 ± 0.16 ^b^	8.97 ± 0.08 ^b^	9.36 ± 0.05 ^a^
C16:1	0.13 ± 0.00 ^b^	0.12 ± 0.00 ^b^	0.19 ± 0.00 ^a^	0.09 ± 0.04 ^c^	0.13 ± 0.00 ^b^
C18:0	5.23 ± 0.12 ^a^	4.39 ± 0.05 ^b^	3.65 ± 0.02 ^d^	4.04 ± 0.11 ^c^	3.99 ± 0.01 ^c^
C18:1	19.62 ± 0.20 ^b^	20.13 ± 0.10 ^a^	17.56 ± 0.06 ^c^	16.75 ± 0.38 ^d^	16.93 ± 0.05 ^d^
C18:2	65.55 ± 0.50 ^b^	66.01 ± 0.07 ^b^	68.74 ± 0.09 ^a^	69.00 ± 0.52 ^a^	68.43 ± 0.11 ^a^
C18:3	0.33 ± 0.00 ^b^	0.35 ± 0.01 ^b^	0.43 ± 0.03 ^a^	0.44 ± 0.01 ^a^	0.43 ± 0.01 ^a^
C20:0	0.16 ± 0.00 ^a^	0.12 ± 0.00 ^b^	0.12 ± 0.01 ^b^	0.15 ± 0.00 ^a^	0.15 ± 0.00 ^a^
C22:0	0.05 ± 0.00 ^a^	0.05 ± 0.01 ^a^	0.06 ± 0.01 ^a^	0.05 ± 0.00 ^a^	0.05 ± 0.00 ^a^
C22:1	0.02 ± 0.00 ^a,b^	0.02 ± 0.00 ^a,b^	0.03 ± 0.01 ^a^	0.01 ± 0.00 ^b^	0.01 ± 0.00 ^b^
SFA	13.80 ± 0.29 ^a^	12.77 ± 0.13 ^c,d^	12.58 ± 0.19 ^d^	13.24 ± 0.18 ^b,c^	13.61 ± 0.06 ^a,b^
USFA	83.58 ± 0.25 ^a^	82.46 ± 0.04 ^a,b^	83.45 ± 0.19 ^a^	81.82 ± 0.86 ^b^	82.06 ± 0.38 ^b^
MUFA	19.77 ± 0.20 ^b^	20.27 ± 0.10 ^a^	17.78 ± 0.05 ^c^	16.85 ± 0.37 ^d^	17.07 ± 0.05 ^d^
PUFA	65.88 ± 0.50 ^b^	66.37 ± 0.08 ^b^	69.17 ± 0.12 ^a^	69.44 ± 0.05 ^a^	68.86 ± 0.10 ^a^
PUFA_ω-6_/PUFA_ω-3_	198.93	188.15	159.61	155.29	160.14

Results are presented as mean ± standard deviation (*n* = 2). Values followed by the same letter in a row are not different at the 95.0% confidence level (One-way ANOVA with Fisher’s LSD). N.D.: not detected.

**Table 3:** Indicator of nutritional quality of grape oils.

	White Grapes			Red Grapes	
	PF	PX	MA	TM	TR
Grape Pomace Oils					
COX values	7.02 ± 0.05 ^c^	6.87 ± 0.08 ^d^	7.36 ± 0.04 ^a^	7.15 ± 0.02 ^b^	7.24 ± 0.02 ^a,b^
USFA/SFA	5.13 ± 0.05 ^b^	6.04 ± 0.06 ^a^	4.79 ± 0.15 ^c^	4.78 ± 0.00 ^c^	5.11 ± 0.06 ^b^
PUFA/SFA	3.97 ± 0.05 ^b,c^	4.73 ± 0.05 ^a^	3.82 ± 0.13 ^c^	3.82 ± 0.00 ^c^	4.10 ± 0.05 ^b^
AI	0.125 ± 0.002 ^c^	0.112 ± 0.002 ^d^	0.149 ± 0.007 ^a,b^	0.157 ± 0.000 ^a^	0.141 ± 0.004 ^b^
TI	0.34 ± 0.01 ^b^	0.30 ± 0.00 ^c^	0.30 ± 0.01 ^c^	0.35 ± 0.00 ^a^	0.33 ± 0.00 ^b^
H/H	8.31 ± 0.13 ^b^	9.45 ± 0.13 ^a^	7.35 ± 0.31 ^c,d^	6.93 ± 0.06 ^d^	7.53 ± 0.22 ^c^
Grape Seed Oils					
COX values	7.02 ± 0.05 ^b^	7.08 ± 0.01 ^b^	7.35 ± 0.02 ^a^	7.37 ± 0.05 ^a^	7.31 ± 0.01 ^a^
USFA/UFA	6.21 ± 0.15 ^d^	6.78 ± 0.08 ^a,b^	6.92 ± 0.12 ^a^	6.52 ± 0.10 ^b,c^	6.31 ± 0.03 ^c,d^
PUFA/SFA	4.78 ± 0.14 ^c^	5.20 ± 0.06 ^b^	5.50 ± 0.09 ^a^	5.25 ± 0.11 ^b^	5.06 ± 0.03 ^b^
AI	0.100 ± 0.002 ^c,d^	0.097 ± 0.001 ^d^	0.103 ± 0.002 ^b,c^	0.106 ± 0.001 ^b^	0.112 ± 0.001 ^a^
TI	0.31 ± 0.01 ^a^	0.28 ± 0.00 ^c,d^	0.28 ± 0.01 ^d^	0.29 ± 0.00 ^b.c^	0.30 ± 0.00 ^a,b^
H/H	10.23 ± 0.25 ^a,b^	10.54 ± 0.14 ^a^	9.92 ± 0.21 ^b,c^	9.56 ± 0.09 ^c^	9.11 ± 0.06 ^d^

Results are presented as mean ± standard deviation (*n* = 2). Values followed by the same letter in a row are not different at the 95.0% confidence level (One-way ANOVA with Fisher’s LSD).

**Table 4:** Tocopherol contents and vitamin E activity of the by-product oils studied. Tocopherol content expressed as mg/kg by-product oil, and vitamin E activity expressed as α-tocopherol equivalent (mg/kg by-product oil).

	White Grapes			Red Grapes	
	PF	PX	MA	TM	TR
Grape Pomace Oil					
α-Tocopherol	1570.1 ± 73.7 ^a^	179.1 ± 11.6 ^d^	367.8 ± 56.6 ^c^	543.5 ± 18.2 ^b^	647.4 ± 5.6 ^b^
β-Tocopherol	N.D.	N.D.	N.D.	N.D.	N.D.
γ-Tocopherol	37.3 ± 0.7 ^b^	35.0 ± 3.0 ^b^	19.5 ± 0.7 ^c^	61.8 ± 2.7 ^a^	37.3 ± 1.8 ^b^
δ-Tocopherol	79.1 ± 3.1 ^a^	14.8 ± 2.3 ^d^	41.6 ± 4.4 ^b^	22.9 ± 0.5 ^c^	21.9 ± 0.2 ^c^
Share of α-Toc. (%)	93.1 ± 0.4 ^a^	78.2 ± 0.9 ^c^	85.7 ± 0.9 ^b^	86.5 ± 0.1 ^b^	91.6 ± 0.3 ^a^
Vitamin E activity	1574.6 ± 73.7 ^a^	182.7 ± 11.9 ^d^	370.2 ± 56.7 ^c^	549.9 ± 18.5 ^b^	651.4 ± 5.4 ^b^
Total tocopherol	1686.4 ± 71.3 ^a^	228.9 ± 12.3 ^d^	428.9 ± 61.7 ^c^	628.2 ± 20.5 ^b^	706.6 ± 3.5 ^b^
Grape Seed Oil					
α-Tocopherol	193.1 ± 1.4 ^a^	188.9 ± 5.4 ^a^	172.4 ± 17.3 ^a^	125.1 ± 12.3 ^b^	114.8 ± 12.3 ^b^
β-Tocopherol	N.D.	N.D.	N.D.	N.D.	N.D.
γ-Tocopherol	44.7 ± 0.8 ^b^	38.6 ± 2.4 ^b,c^	25.6 ± 0.3 ^c^	72.1 ± 8.0 ^a^	73.3 ± 10.3 ^a^
δ-Tocopherol	10.2 ± 0.5 ^b^	N.D.	N.D.	11.9 ± 1.0 ^a^	8.1 ± 0.8 ^c^
Share of α-Toc. (%)	77.9 ± 0.3 ^c^	83.0 ± 0.5 ^b^	87.0 ± 1.0 ^a^	59.8 ± 0.4 ^d^	58.6 ± 0.7 ^d^
Vitamin E activity	197.7 ± 1.5 ^a^	192.7 ± 5.6 ^a^	174.9 ± 17.4 ^a^	132.4 ± 13.1 ^b^	122.2 ± 13.4 ^b^
Total tocopherol	248.0 ± 2.7 ^a^	227.5 ± 7.8 ^a,b^	198.0 ± 17.6 ^b^	209.1 ± 19.4 ^a,b^	196.2 ± 23.5 ^b^

Results are presented as mean ± standard deviation (*n* = 2). Values followed by the same letter in a row are not different at the 95.0% confidence level (One-way ANOVA with Fisher’s LSD). N.D.: not detected.

## Data Availability

Not applicable.

## Sample Availability

Samples of the compounds are available from the authors.

## References

[1] Nogala-Kalucka M., Rudzinska M., Zadernowski R., Siger A., Krzyzostaniak I. (2010). Phytochemical content and antioxidant properties of seeds of unconventional oil plants. J. Am. Oil Chem. Soc..

[2] Daud N.M., Putra N.R., Jamaludin R., Md Norodin N.S., Sarkawi N.S., Hamzah M.H.S., Mohd Nasir H., Abang Zaidel D.N., Che Yunus M.A., Md Salleh L. (2022). Valorisation of plant seed as natural bioactive compounds by various extraction methods: A review. Trends Food Sci. Technol..

[3] Beres C., Costa G.N.S., Cabezudo I., da Silva-James N.K., Teles A.S.C., Cruz A.P.G., Mellinger-Silva C., Tonon R.V., Cabral L.M.C., Freitas S.P. (2017). Towards integral utilization of grape pomace from winemaking process: A review. Waste Manag..

[4] Fernandes L., Casal S., Cruz R., Pereira J.A., Ramalhosa E. (2013). Seed oils of ten traditional Portuguese grape varieties with interesting chemical and antioxidant properties. Food Res. Int..

[5] Hassanein M.M.M., Abedel-Razek A.G. (2009). Chromatographic quantitation of some bioactive minor components in oils of wheat germ and grape seeds produced as by-products. J. Oleo Sci..

[6] Ferreira S.M., Santos L. (2022). A potential Valorization Strategy of Wine Industry By-Products and Their Application in Cosmetics—Case study: Grape Pomace and Grapeseed. Molecules.

[7] Pardo J.E., Fernández E., Rubio M., Alvarruiz A., Alonso G.L. (2009). Characterization of grape seed oil from different grape varieties (*vitis vinifera*). Eur. J. Lipid Sci. Technol..

[8] Nuchdang S., Phruetthinan N., Paleeleam P., Domrongpokkaphan V., Chuetor S., Chirathivat P., Phalakornkule C. (2022). Soxhlet, microwave-assisted, and room temperature liquid extraction of oil and bioactive compounds from palm kernel cake using isopropanol as solvent. Ind. Crops Prod..

[9] Drosou C., Kyriakopoulou K., Bimpilas A., Tsimogiannis D., Krokida M. (2015). A comparative study on different extraction techniques to recover red grape pomace polyphenols from vinification byproducts. Ind. Crops Prod..

[10] Crews C., Hough P., Godward J., Brereton P., Lees M., Guiet S., Winkelmann W. (2006). Quantitation of the main constituents of some authentic grape-seed oils of different origin. J. Agric. Food Chem..

[11] Oomah B.D., Liang J., Godfrey D., Mazza G. (1998). Microwave Heating of Grapeseed: Effect on Oil Quality. J. Agric. Food Chem..

[12] Sabir A., Unver A., Kara Z. (2012). The fatty acid and tocopherol constituents of the seed oil extracted from 21 grape varieties (Vitis spp.). J. Sci. Food Agric..

[13] Tagkouli D., Tsiaka T., Kritsi E., Soković M., Sinanoglou V.J., Lantzouraki D.Z., Zoumpoulakis P. (2022). Towards the Optimization of Microwave-Assisted Extraction and the Assessment of Chemical Profile, Antioxidant and Antimicrobial Activity of Wine Lees Extracts. Molecules.

[14] Settar Unal M., Gundesli M.A., Ercisli S., Kupe M., Assouguem A., Ullah R., Almeer R., Najda A. (2022). Cultivar Differences on Nutraceuticals of Grape Juices and Seeds. Horticulturae.

[15] Lutterodt H., Slavin M., Whent M., Turner E., Yu L. (2011). (Lucy) Fatty acid composition, oxidative stability, antioxidant and antiproliferative properties of selected cold-pressed grape seed oils and flours. Food Chem..

[16] Matthäus B. (2008). Virgin grape seed oil: Is it really a nutritional highlight?. Eur. J. Lipid Sci. Technol..

[17] Tangolar S.G., Özoǧul Y., Tangolar S., Torun A. (2009). Evaluation of fatty acid profiles and mineral content of grape seed oil of some grape genotypes. Int. J. Food Sci. Nutr..

[18] Bail S., Stuebiger G., Krist S., Unterweger H., Buchbauer G. (2008). Characterisation of various grape seed oils by volatile compounds, triacylglycerol composition, total phenols and antioxidant capacity. Food Chem..

[19] Mišurcová L., Ambrožová J., Samek D. (2011). Seaweed lipids as nutraceuticals. Adv. Food Nutr. Res..

[20] Orsavova J., Misurcova L., Ambrozova J., Vicha R., Mlcek J. (2015). Fatty Acids Composition of Vegetable Oils and Its Contribution to Dietary Energy Intake and Dependence of Cardiovascular Mortality on Dietary Intake of Fatty Acids. Int. J. Mol. Sci..

[21] Vujasinovic V.B., Bjelica M.M., Čorbo S.C., Dimic S.B., Rabrenovic B.B. (2021). Characterization of the chemical and nutritive quality of coldpressed grape seed oils produced in the Republic of Serbia from different red and white grape varieties. Grasas Aceites.

[22] Hashempour-Baltork F., Torbati M., Azadmard-Damirchi S., Savage G.P. (2018). Chemical, rheological and nutritional characteristics of sesame and olive oils blended with linseed oil. Adv. Pharm. Bull..

[23] Dimić I., Teslić N., Putnik P., Kovačević D.B., Zeković Z., Šojić B., Mrkonjić Ž., Čolović D., Montesano D., Pavlić B. (2020). Innovative and conventional valorizations of grape seeds from winery by-products as sustainable source of lipophilic antioxidants. Antioxidants.

[24] Ferreira R., Lourenço S., Lopes A., Andrade C., Câmara J.S., Castilho P., Perestrelo R. (2021). Evaluation of fatty acids profile as a useful tool towards valorization of by-products of agri-food industry. Foods.

[25] Ulbricht T.L.V., Southgate D.A.T. (1991). Coronary heart disease: Seven dietary factors. Lancet.

[26] Tangolar S.G.S., Özogul F., Tangolar S.G.S., Yağmur C., Yaĝmur C. (2011). Tocopherol content in fifteen grape varieties obtained using a rapid HPLC method. J. Food Compos. Anal..

[27] dos Santos Freitas L., Jacques R.A., Richter M.F., da Silva A.L., Caramão E.B. (2008). Pressurized liquid extraction of vitamin E from Brazilian grape seed oil. J. Chromatogr. A.

[28] Gliszczyńska-Świgło A., Sikorska E. (2004). Simple reversed-phase liquid chromatography method for determination of tocopherols in edible plant oils. J. Chromatogr. A.

[29] Agostini F., Bertussi R.A., Agostini G., Atti dos Santos A.C., Rossato M., Vanderlinde R. (2012). Supercritical Extraction from Vinification Residues: Fatty Acids, α-Tocopherol, and Phenolic Compounds in the Oil Seeds from Different Varieties of Grape. Sci. World J..

[30] Rupérez F.J., Martín D., Herrera E., Barbas C. (2001). Chromatographic analysis of α-tocopherol and related compounds in various matrices. J. Chromatogr. A.

[31] Bustamante-Rangel M., Delgado-Zamarreño M.M., Sánchez-Pérez A., Carabias-Martínez R. (2007). Determination of tocopherols and tocotrienols in cereals by pressurized liquid extraction–liquid chromatography–mass spectrometry. Anal. Chim. Acta.

[32] Nagy K., Courtet-Compondu M.-C., Holst B., Kussmann M. (2007). Comprehensive Analysis of Vitamin E Constituents in Human Plasma by Liquid Chromatography−Mass Spectrometry. Anal. Chem..

[33] Schwartz H., Ollilainen V., Piironen V., Lampi A.-M.M. (2008). Tocopherol, tocotrienol and plant sterol contents of vegetable oils and industrial fats. J. Food Compos. Anal..

[34] Qureshi A.A., Mo H., Packer L., Peterson D.M. (2000). Isolation and identification of novel tocotrienols from rice bran with hypocholesterolemic, antioxidant, and antitumor properties. J. Agric. Food Chem..

[35] Wen X., Zhu M., Hu R., Zhao J., Chen Z., Li J., Ni Y. (2016). Characterisation of seed oils from different grape cultivars grown in China. J. Food Sci. Technol..

[36] Demirtas I., Pelvan E., Ozdemir I.S., Alasalvar C., Ertas E. (2013). Lipid characteristics and phenolics of native grape seed oils grown in Turkey. Eur. J. Lipid Sci. Technol..

[37] Lachman J., Hejtmánková A., Táborský J., Kotíková Z., Pivec V., Střalková R., Vollmannová A., Bojňanská T., Dědina M. (2015). Evaluation of oil content and fatty acid composition in the seed of grapevine varieties. LWT—Food Sci. Technol..

[38] Górnaś P., Siger A., Segliņa D. (2013). Physicochemical characteristics of the cold-pressed Japanese quince seed oil: New promising unconventional bio-oil from by-products for the pharmaceutical and cosmetic industry. Ind. Crops Prod..

[39] Dabetic N.M., Todorovic V.M., Djuricic I.D., Antic Stankovic J.A., Basic Z.N., Vujovic D.S., Sobajic S.S. (2020). Grape Seed Oil Characterization: A Novel Approach for Oil Quality Assessment. Eur. J. Lipid Sci. Technol..

[40] Göktürk Baydar N., Özkan G. (2006). Tocopherol contents of some Turkish wine by-products. Eur. Food Res. Technol..

[41] Ribeiro L.F., Ribani R.H., Francisco T.M.G., Soares A.A., Pontarolo R., Haminiuk C.W.I. (2015). Profile of bioactive compounds from grape pomace (*Vitis vinifera* and *Vitis labrusca*) by spectrophotometric, chromatographic and spectral analyses. J. Chromatogr. B. Analyt. Technol. Biomed. Life Sci..

[42] El Gengaihi S., Ella F.M.A., Hassan E.M., Shalaby E.A., Baker D.H.A. (2013). Phytochemical Investigation and Radical Scavenging Activity of Wastes of Some Grape Varieties Grown in Egypt. Glob. J. Pharmacol..

[43] Gülcü M., Uslu N., Özcan M.M., Gökmen F., Özcan M.M., Banjanin T., Gezgin S., Dursun N., Geçgel Ü., Ceylan D.A. (2018). The investigation of bioactive compounds of wine, grape juice and boiled grape juice wastes. J. Food Process. Preserv..

[44] Puertas García B., Cruz García S., Lara Benítez M., García de Luján A. (2000). La tintilla de Rota: Variedad tradicional de la provincia de Cádiz y sus perspectivas de futuro. Rev. Of. la Fed. Esp. Asoc. Enol..

[45] Yuan C., Xie Y., Jin R., Ren L., Zhou L., Zhu M., Ju Y. (2017). Simultaneous Analysis of Tocopherols, Phytosterols, and Squalene in Vegetable Oils by High-Performance Liquid Chromatography. Food Anal. Methods.

[46] Aksoz E., Korkut O., Aksit D., Gokbulut C. (2020). Vitamin E (α-, β + γ- and δ-tocopherol) levels in plant oils. Flavour Fragr. J..

[47] Mikołajczak N., Tańska M. (2022). Effect of initial quality and bioactive compounds content in cold-pressed flaxseed oils on oxidative stability and oxidation products formation during one-month storage with light exposure. NFS J..

[48] Carmona-Jiménez Y., García-Moreno M.V., García-Barroso C. (2018). Effect of Drying on the Phenolic Content and Antioxidant Activity of Red Grape Pomace. Plant Foods Hum. Nutr..

[49] AOAC, AOAC 920.85-1920 (1990). Fat (crude) or ether extract in flour. Methods of the Association of Official Analytical Chemists.

[50] Gallart M., Francioli S., Viu-Marco A., López- Tamames E., Buxaderas S. (1997). Determination of free fatty acids and their ethyl esters in musts and wines. J. Chromatogr. A.

[51] Gómez M.E., Igartuburu J.M., Pando E., Luis F.R., Mourente G., Rodríguez Luis F., Mourente G., Rodríguez-Luis F., Mourente G. (2004). Lipid Composition of Lees from Sherry Wine. J. Agric. Food Chem..

[52] Fatemi S.H., Hammond E.G. (1980). Analysis of oleate, linoleate and linolenate hydroperoxides in oxidized ester mixtures. Lipids.

[53] Hassanien M.M.M., Abdel-Razek A.G., Rudzińska M., Siger A., Ratusz K., Przybylski R. (2014). Phytochemical contents and oxidative stability of oils from non-traditional sources. Eur. J. Lipid Sci. Technol..

[54] Lee J., Suknark K., Kluvitse Y., Phillips R.D., Eitenmiller R.R. (1999). Rapid liquid chromatographic assay of vitamin E and retinyl palmitate in extruded weaning foods. J. Food Sci..

[55] McLaughlin P.J., Weihrauch J.L. (1979). Vitamin E content of foods. J. Am. Diet. Assoc..

[56] Bada J.C., León-Camacho M., Copovi P., Alonso L. (2015). Characterization of grape seed oil from wines with protected denomination of origin (PDO) from Spain. Grasas Aceites.

[57] Bondioli P., Folegatti L., Rovellini P. (2020). Oils rich in alpha linolenic acid: Chemical composition of perilla (*Perilla frutescens*) seed oil. OCL.

[58] Nosratpour M., Farhoosh R., Sharif A. (2017). Quantitative Indices of the Oxidizability of Fatty Acid Compositions. Eur. J. Lipid Sci. Technol..

[59] Szpunar-Krok E., Wondołowska-Grabowska A. (2022). Quality Evaluation Indices for Soybean Oil in Relation to Cultivar, Application of N Fertiliser and Seed Inoculation with *Bradyrhizobium japonicum*. Foods.

[60] Department of Health (1994). Nutritional Aspects of Cardiovascular Disease. Report of the Cardiovascular Review Group Committee on Medical Aspects of Food Policy. Reports Health Soc. Subj..

[61] Köseoğlu O., Sevim D., Kadiroğlu P. (2019). Effects of Filtration on the Quality Properties of Extra Virgin Olive Oils during Storage. J. Am. Oil Chem. Soc..

